# An exploration of strategies used by older people to obtain information about health‐ and social care services in the community

**DOI:** 10.1111/hex.12408

**Published:** 2015-09-08

**Authors:** Margaret Mc Grath, Kathleen Clancy, Anne Kenny

**Affiliations:** ^1^ School of MedicineTrinity College DublinThe University of Dublin (Singapore)Singapore; ^2^ King's College Hospital NHS Foundation TrustLondonSE5 9RSUnited Kingdom; ^3^ Senior Support ServicesCOPE GalwayGalwayIreland

**Keywords:** health literacy, information seeking, Ireland, older people

## Abstract

**Purpose:**

To explore the strategies used by older people living in Ireland to obtain information about community health and social services.

**Methods:**

A qualitative exploratory design was used. Focus groups (*n* = 3) were conducted with community dwelling older people (*n* = 17). A series of vignettes were used to guide discussion regarding hypothetical situations that approximated real‐life scenarios for older people. Data were transcribed verbatim and analysed using content analysis.

**Results:**

Obtaining information about community health and social services is an ongoing process that requires continuous commitment by older adults. Key strategies which emerged from the data included (i) taking a proactive stance towards accessing health information, (ii) making use of personal networks in your community and (iii) developing ‘insider’ knowledge.

**Conclusion:**

Older people in this study had a proactive approach to obtaining health information and identified the importance of taking responsibility for managing their own needs. Despite this, obtaining basic information about community health and social services was a challenging and time‐consuming process. Future research should focus on developing health literacy interventions that build upon and expand the strategies currently used by older people.

## Introduction

### Health literacy

Health literacy, a concept first introduced in the 1970s,^1^ plays an important role in rehabilitation and health care.^2^ Nutbeam^3^ notes that high levels of health literacy have benefits for both individuals and broader society. Sufficient levels of health literacy enable individuals to understand the factors that influence their health and to take responsibility for addressing these factors. This leads to improved knowledge of health services, compliance with prescribed health interventions, an increased capacity to act independently, improved motivation and self‐confidence to act on health issues and increased resilience to social and economic adversity.^4^ For the wider community, high levels of individual health literacy increase participation in population health programmes, support community empowerment and enhance capacity to address social and economic determinants of health.^3^

Despite increased interest in health literacy, there is no universally accepted definition of the concept.^2^ The World Health Organization suggests that health literacy refers to the ability to engage with health information and health services.^5^ In this way, health literacy can be considered as a process consisting of the following three stages, obtaining, understanding and making use of information in a way that is meaningful to the individual.^6^, ^7^, ^8^

Sorensen *et al*.^2^ propose a conceptual model of health literacy which requires health literate individuals to possess four types of competence (i) access – the ability to seek, find and obtain health information; (ii) understanding – the ability to comprehend health information which is accessed; (iii) appraisal – the capacity to interpret, judge and evaluate the information which has been obtained; and (iv) application – the ability to communicate and use the information obtained to make decisions about health. According to Sorensen *et al*.,^2^ each of these competences represents a critical aspect of health literacy and possession of all four competences will enable an individual to navigate three dimensions of the health continuum: being ill or as a patient in a health‐care setting, as a person at risk of disease in the disease prevention system, and as a citizen who participates in health promotion efforts in the community.^2^

### Health literacy and older people

As a group older people (those aged 60 years and upwards) frequently participate in all three dimensions of health described by Sorensen *et al*.^2^ Despite being frequent consumers of health‐care services, older people often possess lower levels of health literacy when compared with other groups in society.^9^, ^10^ A recent survey of health literacy in Europe found a negative correlation between age and health literacy in seven of the eight countries surveyed.^11^ Similar results have been found in the United States, England and Canada where between 29 and 88% of older people were found to have below basic levels of health literacy, that is having no more than the most basic concrete literacy skills.^12^, ^13^, ^14^ A number of reasons have been offered to explain why older people appear to be more vulnerable to low levels of health literacy when compared with other groups in the population. As people grow older, they may lose access to both informal and formal sources of information.^15^ At the same time, older people frequently have complex health needs and are thus required to access, appraise and apply information from multiple service providers and health‐care professionals to make appropriate decisions about their health care.^16^, ^17^ Research suggests that older adults have lower than expected awareness of health‐ and social care services available to support them^18^ which may result in older people failing to make timely contact with preventative health‐ and social care services. In addition, advances in technology mean that much of this information is available online^16^, ^17^ and while an increasing number of older people are users of Internet technology^19^ there continues to be a significant age divide with much higher usage among younger people when compared with older people.^20^ Furthermore, among older adults in Europe, Internet usage is strongly linked to socio‐economic factors with only 15% of older people in lower income groups having used the Internet.^19^

In addition to being more prevalent among older people, lower levels of health literacy may also disproportionately affect the health of older people because older people have to deal with higher levels of chronic disease and age‐related cognitive decline.^10^, ^13^ Bostock^13^ in a study of functional health literacy among older people in the United Kingdom found the low health literacy was associated with a 75% increased risk of mortality compared with high levels of health literacy, while in the United States, Wolf, Gazmararian and Baker^21^ demonstrated that individuals with inadequate health literacy had poorer physical function and mental health.

### Improving health literacy among older people

Despite recognition of the impact of health literacy on health and quality of life for older people, limited attention has been paid to developing health literacy interventions for older people.^22^ Programmes that do exist, appear to have focused almost entirely on use of the Internet and for the most part have been based in North America.^22^ Although useful, such programmes may have limited success where large proportions of older people do not have access to or use Internet technology.^20^ Rather there is a need for health literacy programmes for older people which build upon individuals’ existing skills in each of the four competency areas identified in Sorensen *et al*.'s^2^ model of health literacy, that is accessing, understanding, appraisal and application of knowledge.

A first step in developing a health literacy programme for older adults that builds upon prior knowledge is to consider what strategies older use in each of the four stages of health literacy. At present limited information is available regarding how older adults go about accessing, understanding, appraising or applying knowledge related to their health. This research attempts to begin to address this gap through exploring how older people living in the community in Galway Ireland access, understand, appraise and apply information about health‐ and social services in their community.

## Methods

### Participants

Participants were purposively selected from older adults living in the community who had previous experience of using health‐ and social care services. Participants were recruited through Cope Galway Senior Support Services, Galway. Cope Galway is a voluntary organization which provides a range of services and supports to older people with an aim to enable them to remain living independently at home and reduce the risk of isolation. Research participants were included if they (i) were ≥60 years of age, (ii) were living in a private home in the community in Galway city, that is not living in a nursing home or assisted living facility, and (iii) had previous experience of accessing community health or social services. Additional criteria included the ability to attend a focus group meeting on the selected date. Focus group meetings were held in locations which were familiar to participants, for example community and day centres.

### Data collection procedures

Three focus groups, with three different groups of older people, each approximately 90 min in length were conducted. Groups were moderated by one of the researchers (KC) with a second researcher (MMCG) serving as note‐taker. All of the focus groups were recorded using a digital audio recorder. Each of the focus groups followed the same structure and began with clarification of the purpose of the project and issues relating to confidentiality. The moderator then used a semi‐structured focus group protocol including open‐ended questions and vignettes, adapted from Ploeg *et al*.,^23^ to elicit group participation and discussion relating to the topic area (Table 1). The original study describes 12 possible vignettes from which four were used in each focus group. The choice of vignettes was made in collaboration with older adults living in the community and not involved in this research who were asked to comment on the validity of the vignettes for older people in Ireland. Following these discussions, minor changes were made to the existing vignettes so that the language used reflected an Irish context.

**Table 1:** Vignettes

Vignette title	Vignette
Grief recovery	Your spouse died 2 years ago. You spend a lot of time watching game shows and soap operas. Your family expects you to get on with life. You wish you had someone to talk to
Leisure	You are single and recently retired. You have never had time to pursue any leisure activities. You are having trouble filling your time
Maintaining your independence	You have poor health and are no longer able to do your shopping, housework or gardening. Your family members are busy and you don't want to bother them
Transportation	You have to go for chemotherapy at the hospital several times per week. Your family and friends are unable to help you. You cannot afford a taxi and are too weak to take the public transport

Reproduced with permission from Denton, Ploeg, Tindale, Hutchison, Brazil, Akhtar‐Danesh *et al*. (2008).

### Ethical considerations

The research ethics committee at the National University of Ireland, Galway, approved this study. All participants were provided with written and oral information concerning the aim and methods of the study. Emphasis was placed on the voluntary nature of participation in the study, and participants were assured that all information would remain anonymous. Prior to participation in the focus group, all participants signed consent forms indicating their willingness to participate in the study.

### Data analysis

The recordings from each of the focus groups were transcribed verbatim, and the transcribed data were analysed using content analysis as described by Graneheim and Lundman.^24^ The content analysis process began with several readings of the focus group transcriptions. At this stage, the aim of the analysis was to become familiar with the overall content of the group discussion. Using NVivo, the first author coded the transcript material so that descriptive labels were generated for each sentence from the transcripts. These codes were then grouped into categories that shared common features. From these categories, larger groups of categories and finally themes emerged. At each stage of the analysis process, the credibility of the findings was enhanced by returning to the original transcripts and through discussion with the other authors (AK and KC).

## Results

A total of 17 older people (eight males, nine females) participated in the study. All of the participants were living independently in the community and had previous experience of using local health and social services. Participants’ ages ranged from 62 to 85 with a mean age of 78.5 years. Data from the focus groups yielded rich data relating to older peoples’ experiences and strategies for accessing information about health and social services. Participants generally reported low levels of knowledge of local health and social services and perceived that it was difficult to access this knowledge. The analysis revealed three main themes relating to strategies used by participants to access information about services (i) taking a proactive stance towards accessing health information, (ii) making use of personal networks in your community and (iii) developing ‘insider’ knowledge. Each strategy was supported by a number of actions taken by participants, and for the majority of participants, all strategies were used on an ongoing basis as they sought to manage their everyday health‐ and social care needs.

### Taking a proactive stance towards accessing health information

The majority of participants described themselves as adopting a proactive stance towards accessing information about health and social services. As a result, they were constantly alert for opportunities to learn about services either through talking to other people or observing developments in their local community. One participant described how she was told about a service that offered support to older people who were undertaking home maintenance tasks. She had telephoned this service but was unable to find out how to access the support she needed. Despite this she notes that

P8 I've found what I can do and one day I was sitting observing, not gossiping just observing and I saw a [names organisation] van so I went out and spoke to the man [a member of staff from the organisation] and it isn't [names a second organisation] it's what is the name does anybody know ? I've got it in my notebook anyway, its [names organisation] or something like that for help it's got a different phone number and they will put you on a list and you'll be seen to within a few days

This type of experience resonated with other focus group participants and many noted that there is a need for determination when seeking information that is not always readily available or accurate. As one participant commented you need to have ‘will power’ (P9) while others noted that the only way to get information is by ‘sticking to the phone’ (P13) and that ‘a lot depends on the person themselves you know easy to get on with and such, the help will come to them’ (P1).

This proactive stance appeared to emerge from a belief that individuals have a personal responsibility to identify their own health‐care needs and resources. As participants in one focus group noted

P2 You have to ask. You have to be forward. They won't come to you.

P5 Yes, nobody's going to come into you personally.

While others commented that services frequently forget to keep older people informed of changes and developments.

P16 you're told nothing … so I mean these kind of things [referring to a health clinic which had moved location] if there is a change, if there's a change in location, if there's a change in their.. if they are changing from three to six months [referring to frequency of appointments] …I haven't been informed of anything

### Making use of personal networks in your community

Participants described using personal networks to obtain information. These networks included family, neighbours and trusted public figures in the community, for example local librarians, pharmacists, staff in the citizen's advice bureau and local police.

P4 Someone across the road told me [about a day care service]

P1 You had a chat with some hackney [taxi] driver that you'd know and see is there any scheme. A lot of those guys would know if there was any assistance available towards the cost of transport

P17 the guards [police]

Participants who felt most confident about accessing relevant information reported that these networks had been built up over a number of years and reflect ongoing involvement and participation in their communities. Other participants had less personal contacts within their communities and described feeling unsure of how to develop these networks.

P17 I do be saying I'm stuck in a limbo at the moment because my back is so bad and nobody is helping me do you know …so there's a lot of us out there that's stuck in a limbo, no door out do you know?

### Developing ‘insider’ knowledge

Most of the participants in our study were of the opinion that obtaining information about relevant health and social services involved a period during which the older adult had to negotiate to get ‘inside’ the system. The process of ‘getting in’ could often be difficult and involve multiple attempts, but once the individual was ‘in’, there was a general perception that future information would be easier to obtain. As one participant notes.

P15 [once] you get into the system at all you're grand

To get ‘in’ participants sought access to those who were perceived to have ‘insider’ knowledge. Such ‘insiders’ were people who worked within the health‐ and social care system but were perceived as being more accessible to participants than health‐ and social care professionals. Examples of ‘insiders’ included hospital porters; clerical staff working in clinics; cleaning staff; catering staff; and security guards. Participants described a balancing act between asking for assistance from knowledgeable ‘insiders’ and ensuring that they were not perceived as too demanding by these insiders.

P11 and very often if you have a very keen interest, the receptionist or somebody like that would be happy to give advice. Then again, you'd have to use a bit of common sense and don't be bothering them when they're busy and maybe wait until the mad rush is over and then go over and have a chat with them. That would be one way. It's amazing what you can pick up that way

This ‘insider’ knowledge was perceived to be more useful than ‘official’ information provided by these services which was either too complicated to understand, not personalized to the needs of the individual or out of date.

P5 sometimes, of course, to get, like, you find an awful amount of web pages which are not up to date

And

P17 the consultation with the doctor or nurse, sometimes there's a lot of information thrown at you in a very short space of time

## Discussion

The overall aim of this research was to explore strategies used by older people living in the community to access and obtain information about local health and social services. The findings have generated a number of key insights for service providers and researchers and suggest that further work is required to ensure that older adults can learn about (and ultimately access) appropriate community health and social services.

The overall findings regarding the challenging nature of obtaining information about health and social services reflects previous research which suggests that older people are particularly vulnerable to having low levels of health literacy and as a result are likely to experience challenges in obtaining relevant information.^9^, ^10^, ^12^, ^13^, ^14^ Despite this vulnerability, it appears that many of the participants in this study were attempting to tackle these challenges and had developed a repertoire of actions that were directed towards obtaining the information they needed. Many of these strategies appeared to be reliant on active participation in the local community and reflect what Wagner, Messick and Spratt^25^ describe as distributed literacy that is where individuals each possessing some aspect of literacy combine their efforts so as to function as more fully literate. Similar findings have been reported by Edwards, Wood, Davies and Edwards^26^ among people living with long‐term health conditions and Papen^27^ suggests that viewed as a distributed resource health literacy becomes reliant not just on the individuals skills but also upon their available social supports. The results of the current study support this understanding of health literacy and for those participants who did not have a strong connection to their local community obtaining information appeared to be more difficult. There is increased recognition that social isolation adversely affects long‐term health outcomes for older people^28^ and certainly in this study it appeared that participants who were more socially isolated did not have the same capability to respond to the challenge of obtaining health information. Given that at present over 28% of older people in Ireland live alone,^29^ this finding suggests that future interventions may need to be directed at those older people most at risk of isolation or who live alone.

Additional questions have been raised by the findings in relation to the role of community‐based services which have a remit to support older people to access information about their health‐care needs. The majority of participants in this study did not expect that information would be provided by service providers and instead had adopted a proactive stance towards seeking out information for themselves. This may reflect recognition among older people that health and social services are increasingly moving towards a consumerist model of care^30^ where there is an implicit obligation on individuals to search for information themselves, to understand their rights and responsibilities and to make their own decisions about care.^8^

This strategy may also reflect an awareness of the challenges faced by the health‐ and social care system given the current economic climate in Ireland and ongoing media reports about health‐ and social care staff shortages and service closures.^31^, ^32^ However, while provision of health‐ and social care services for older people is undoubtedly a priority, service providers must also prioritize provision of information about these services. A review of health literacy in Ireland suggests that at a national level, health literacy has been given limited priority by policymakers or service providers^33^ and this is reflected in the behaviours of adults in Ireland with 43% of people reporting that they would only sometimes ask their health‐care professional to clarify information if they did not understand something they had said.^34^ This is unfortunate as among people aged 75 or over, nearly 60% report a chronic illness^35^ therefore access to accurate information and support is relevant to a significant number of older adults in the general population. Previous research has demonstrated that involving people in their own care and improving health literacy levels can result in increased use of preventative services,^36^, ^37^ reduce hospital admissions^37^, ^38^ and ultimately reduce health‐care costs^4^, ^39^.

In the absence of direct information from service providers participants in this study sought out trusted public figures *(*a local librarian, tax drivers, priest and police services), who they perceived as reliable and accessible sources of health information. This finding reflects previous research which has identified that health‐care consumers frequently learn about health‐care in diverse and complex ways following their own logic and often drawing from sources which may not been seen as legitimate by health‐care providers.^40^, ^41^

In the current study, the majority of the public figures who were identified by the older participants in the study do not have an ‘official’ health information remit; however, it seemed that for many of the participants in our study these public figures had sufficient knowledge to resolve everyday problems. Future interventions could perhaps build upon this strategy by including public figures such as police officers, clergy members and librarians. in the development of programmes to increase knowledge of health‐ and social care services. Such an approach would be congruent with the principles of participatory learning^42^ and calls for cross‐sectoral collaboration to support improvements in health literacy.^7^ Indeed, the National Adult Literacy Agency has argued that community‐based education is the most appropriate model to enhance the development of health literacy in Ireland.^33^

Increasingly health and social services providers make information regarding services, clinic times and contact details available via Internet sites.^43^ Although participants in this study were aware of this trend and at times, many did make use of the Internet, for the most part they preferred to receive information from personal sources and local public figures who were perceived as being more robust sources of information. Searching for information on the Internet is complex as it requires a general knowledge of the topic of interest (in this case health and social services for older people), knowledge of hardware and software operations, information seeking skills (how the Internet is organized, how links, search engines and search histories work) and ability to judge the credibility of information sources.^44^ Previous research suggests that for many older adults in Ireland and indeed in Europe internet usage is not universal^19^, ^35^ and among older adults is strongly associated with socio‐economic status.^45^ For this reason, while Internet‐based information may be useful for some older people, service provides must consider the needs on non‐Internet users. Failure to develop alternative information sharing strategies risks increasing health‐care disparities among population groups.

## Limitations

Although this study has generated some interesting findings regarding the actions taken by older people to access, understand, appraise and apply information relating to local health‐ and social care services, there are some limitations which must be considered when interpreting the study findings.

While qualitative research enables researchers to study structures, processes and phenomena which impact on peoples everyday lives the extent to which qualitative research findings can be generalized is not always clear. Our research was conducted with a specific group of older people living in the West of Ireland. Although the focus groups yielded rich data, it may be that the experiences reported by these participants and the strategies they have developed in response to these experiences are unique to the specific and contextual situations of the participants. The findings therefore may or may not be transferable to other older people in Ireland or to older people in other jurisdictions.

There are likely additional meaningful strategies relating to health literacy that were not captured by this report. Still we believe that the results from the current study highlight the importance of considering the approaches taken by older people to manage their own health needs. Focusing on these strategies when developing future interventions to improve health literacy may ensure that such programmes are grounded in a participatory learning based perspective^42^ which seeks to empower people so that they ‘may gain control over their own lives in the context of participating with others to change their social and political realities (p.12)’.^46^

## Conclusion

There is a growing expectation by health‐ and social care service providers that service users are active participants in seeking information and accessing services.^9^, ^30^ In this study, older people demonstrated a proactive approach to obtaining health information and identified the importance of keeping themselves up to date with information that was relevant to their own needs. However, obtaining basic information about health‐ and social care services in their community was a challenging and time consuming process. For health‐ and social care providers, this study provides new insights into the challenges faced by older people and we suggest that future research should focus on using this knowledge to develop health literacy interventions which built upon existing information seeking strategies.

## Funding statement

This study was supported by the Bright Ideas Community‐Engaged Research Initiative, National University of Ireland, Galway.

## Declaration of interest

The authors report no conflict of interests.
